# mTORC1 is also involved in longevity between species

**DOI:** 10.18632/aging.203129

**Published:** 2021-05-29

**Authors:** Gustavo Barja, Reinald Pamplona

**Affiliations:** 1 Faculty of Biology, Complutense University of Madrid, Madrid, Spain; 2 Department of Experimental Medicine, University of Lleida-Lleida Biomedical Research Institute, Lleida, Spain

**Keywords:** longevity, aging rate, Cell Aging Regulating System (CARS), nuclear aging program (AP), mTORC1 complex

Many specific cell afferent signalling pathways like the insulin/IGF-1-like or the mechanistic target of rapamycin (mTOR) pathways contribute to modify longevity by modulating the nuclear aging program (AP) [1,2] target gene expression, which modulates the activity of its various aging effectors [2]. In comparisons between animal species, those with superior longevity have aging effectors with: 1. A low rate of mitROS generation at mitochondrial complex I, specifically at the FeS cluster N1a containing NDUFV2 hydrophilic domain complex I polypeptide [3]. 2. A high rate of base excision repair in mitDNA [4], but not in nuclear DNA. 3. A low abundance of highly unsaturated fatty acids in cell membranes including the mitochondrial ones. 4. Perhaps a low rate of telomere shortening in mitotic tissues as suggested by a single study in mammals plus birds [5]. 5. Differences in metabolomic and lipidomic profiles [6]. Concerning the other putative aging effectors, it is unknown whether autophagy, apoptosis, proteostasis, senescent cells, blood factors responsible for heterochronic parabiosis, pro- and anti-aging effects, inflammaging, and epigenetics, which seem to be relevant for aging within species, show or not also different levels between species. Such research is sorely needed before these can be considered also to contribute to longevity determination between species. The cooperating action of all the known and unknown aging effectors together, expressed at different levels under the control of the nuclear AP, generates the aging rate, fast in short-lived and slow in long-lived animal species.

MTOR is member of an evolutionarily conserved group of serine/threonine kinases highly conserved in eukaryote cells. mTOR is present as two distinct complexes: mTOR complex 1 (mTORC1) and mTOR complex 2 (mTORC2). mTORC1 acts as a network hub monitoring and integrating a broad diversity of extra- and intracellular signals, contributing to regulate cell physiology and metabolism, growth, proliferation and aging, through a wide range of downstream pathways including mRNA translation, protein and lipid biosynthesis, mitochondrial function, autophagy, and stress responses. Downregulation of the mTOR network downstream activity increases longevity in several animal models from yeast to mice, whereas mTOR activation shortens longevity accelerating aging and the appearance of age-related degenerative diseases, including cancer and neurodegeneration [7].

In mammals, mTORC1 is composed by mTOR and its associated proteins Raptor (regulatory associated protein of TOR), mLst8 (mammalian lethal with SEC13 protein 8), PRAS40 (Proline-rich AKT1 substrate of 40 kDa), and Deptor (DEP domain-containing mTOR-interacting protein) [7]. Raptor and PRAS40 are present exclusively in mTORC1. mTOR, Raptor, and mLST8 are core components, and DEPTOR and PRAS40 are inhibitory subunits. FK506 binding protein (FKBP12) is a regulatory subunit of the rapamycin sensitive mTORC1 activity. Extra- and intracellular signals that converge in mTORC1 include amino acids, ATP, glucose, growth factors, hormones, molecules of intermediary metabolism, and oxygen [7]. Among amino acids, arginine, leucine, and methionine cycle metabolites play a relevant role as activators of mTORC1 through their interaction with intracellular mediators like the SAMTOR-GATOR1 complex.

Studies in single species have shown that lowering mTORC1 downstream activity through caloric restriction (CR), genomics modulation, or drugs like the mTORC1 inhibitor rapamycin, increases longevity, CR having the greatest effect. However, the possible existence of relevant differences in mTORC1 and its regulatory components between mammalian species with different longevities has never been investigated. In a recent study [8], we used droplet digital PCR (ddPCR) and western blot methods to measure the steady-state levels of gene expression and protein content of the mTORC1 complex and its regulators in mammalian species with different longevities. Targeted metabolomics was also applied to measure the concentration of mTORC1 activators. Heart tissue of eight mammalian species differing in longevity by more than one order of magnitude —from 3.5 years in mice to 46 years in horses— was studied. The results demonstrated: i) the existence of species-specific differences in gene expression and protein content of mTORC1; ii) that long-lived phenotypes correlate with *low* concentration of mTORC1; iii) the presence of low concentrations of mTORC1 activators and high concentration of mTORC1 inhibitors in long-lived animals, and: iv) that these differences are independent of phylogeny [8]. These observations suggest that downregulation of mTORC1 expression and activity is an adaptation that contributed to modify longevity during mammalian evolution in coordination with the other multiple aging effectors controlled by the nuclear AP within the Cell Aging Regulating System [2].

To the best of our knowledge, that investigation is the first comparative study of gene expression and amount of mTOR proteins and their regulators as a function of species longevity [8]. The positive activators of mTORC1 downstream activity, mTOR, Raptor, arginine, methionine and the methionine-related metabolites, SAM and homocysteine, negatively correlated with longevity. And the negative regulators of mTORC1 activity mTORSer2448 and PRAS40 were positively correlated with longevity. All these changes cooperate working together to decrease mTOR downstream activity. The results showed a highly coherent concerted action of mTOR and its regulators, and strongly suggest that mTORC1 downstream activity also participates in the control of longevity between species. The results suggest that maintaining a low mTORC1 downstream activity during adult life is one among various signals collaborating to increase longevity both within and between species.
